# Workplace violence in primary health care workers: an integrative review

**DOI:** 10.47626/1679-4435-2025-1504

**Published:** 2026-01-02

**Authors:** Ashly-Pradenas Cárcamo, Tatiana-Klijn Paravic

**Affiliations:** 1 Universidad de Concepción, Facultad de Enfermería, Programa de Magíster en Enfermería, Concepción, Región del Biobío, Chile; 2 Universidad de Concepción, Departamento de Fundamentos de Enfermería y Salud Pública, Concepción, Región del Biobío, Chile

**Keywords:** workplace violence, primary health care, health personnel., violencia laboral, atención primaria de salud, personal de salud.

## Abstract

Workplace violence is an important and growing public health problem in primary health care, compromising both the quality of care and workers’ health. However, the available evidence at this level of care remains limited. The objective of this article is to identify and summarize the existing evidence on workplace violence among primary health care workers, considering its prevalence, associated factors, consequences, and preventive measures. An integrative review was conducted using the Web of Science, PubMed, Scopus, SciELO, and Virtual Health Library databases. Quantitative and qualitative studies published in the last 5 years in Spanish, English, or Portuguese were included. Fourteen articles were analyzed. The results showed that verbal workplace violence was the most prevalent form. The most relevant associated factors were being female, working as a nurse or nursing technician, having direct contact with patients, and working shifts. Psychological consequences included feelings of guilt, anxiety, and doubts about professional competence; organizational consequences included increased staff turnover and absenteeism. Preventive measures implemented were scarce and mainly focused on surveillance and communication skills training. The review concludes that verbal workplace violence is highly prevalent and driven by individual, structural, and organizational factors. Its consequences affect both psychological well-being and organizational dynamics, and a substantial gap persists regarding preventive measures.

## Abstract (es)

La violencia laboral es un importante y creciente problema de salud pública en la atención primaria, que compromete tanto la calidad de la atención como la salud de los trabajadores. No obstante, la evidencia sigue siendo limitada en este nivel asistencial. El objetivo del presente artículo es identificar y sintetizar la evidencia disponible sobre violencia laboral en trabajadores de atención primaria de salud, considerando su prevalencia, factores asociados, consecuencias y medidas preventivas. Se realizó una revisión integrativa en las bases de datos Web of Science, PubMed, Scopus, SciELO y Biblioteca Virtual en Salud. Se incluyeron estudios cuantitativos y cualitativos publicados en los últimos 5 años, en español, inglés o portugués. Se analizaron 14 artículos. Los resultados mostraron que la violencia laboral de tipo verbal fue la más prevalente. Los factores asociados más relevantes fueron: ser mujer, desempeñarse como enfermero o técnico en enfermería, mantener contacto directo con usuarios y trabajar en turnos. Las consecuencias psicológicas incluyeron sentimientos de culpa, ansiedad y dudas sobre la competencia profesional; mientras que, a nivel organizacional, se observaron aumento de rotación de personal y ausentismo laboral. Las medidas preventivas implementadas fueron escasas, centradas principalmente en la vigilancia y en el fortalecimiento de competencias comunicacionales de los trabajadores. Se concluye que la violencia laboral verbal es altamente prevalente y se ve favorecida por factores individuales, estructurales y organizacionales. Sus consecuencias afectan tanto el aspecto psicológico como la dinámica organizacional, persistiendo una amplia brecha en relación con las medidas preventivas.

## INTRODUCTION

Workplace violence represents a major public health challenge whose incidence has grown over time.^1^ The International Labor Organization (ILO) defines it as “a set of unacceptable behaviors and practices, or threats of such behaviors and practices, whether manifested once or repeatedly, that aim to cause, or are likely to cause, physical, psychological, sexual, or economic harm”.^2^ Globally, an estimated 23% of the population has experienced some form of workplace violence.^3^ Health care professionals are among the most exposed groups, with a prevalence of 61.9% among nurses and physicians, underscoring the need for an in-depth investigation on this issue and for the adoption of contextualized, evidence-based measures to prevent its occurrence.^4^

In Chile, primary health care (PHC) services are a key component of the health care network, since they serve as the first level of care, or the main “entry point”, for the population into the health care system, and have a strong focus on disease prevention and health promotion. These facilities solve approximately 85% of the population’s most common health problems, generally related to less severe conditions.^5^,^6^ Their teams consist of a wide range of professionals, such as physicians, nurses, midwives, physical therapists, dentists, psychologists, and social workers, as well as administrative, cleaning, and security staff, among others.^7^ Comprehensive protection of workers’ health is essential to ensure the proper functioning of these centers, provide a safe work environment, and contribute both to staff well-being and to the quality of care delivered to the community.^8^

**Figure 1:**
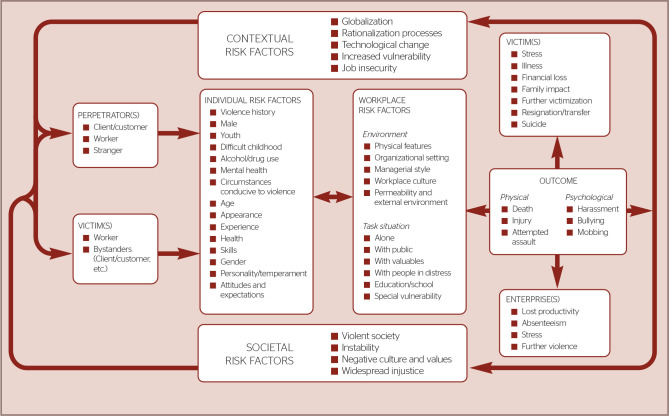
Interactive model of workplace violence by Chappell & Di Martino.

Source: Chappell & Di Martino^9^

Authors such as Chappell & Di Martino have investigated workplace violence, especially in health care settings, and proposed a multifactorial approach known as the “interactive model of workplace violence” to analyze and explain this phenomenon. This model considers multiple risk dimensions that contribute to its origin and development, including personal factors of both the perpetrator and the victim, workplace conditions, and relevant social and contextual factors that support its understanding, prediction, and, ultimately, prevencion.^9^ The Chappell & Di Martino’s interactive model of workplace violence is presented below (Figure 1).

PHC is a high-risk environment for this type of violence, because it is affected by several factors that may compromise both the quality of care and staff well-being. These include service users who experience frustration due to difficulties in accessing services, inadequate care conditions, interaction with patients in critical or high-risk situations, and frequent contact with highly-stressed individuals. Furthermore, excessive workload increases tension or stress among health care workers, contributing to the occurrence of violent episodes in the workplace.^10^

Victims of workplace violence frequently develop adverse health responses, including risk behaviors such as smoking, drinking, and drug use. These effects extend beyond the individual, influencing families, coworkers, and society at large, but also deteriorates their quality of life, compromising their mental health and leading to symptoms such as low self-esteem, anxiety, stress, and even suicidal behavior.^11^,^12^ At the organization level, workplace violence is negatively associated with increased absenteeism and reduced job performance, reflected in lower employee satisfaction and staff retention.^13^

Growing concern over the impacts of workplace violence in health care has encouraged the adoption of worldwide strategies to mitigate the problem. Among these initiatives is the collaborative development of the “Framework Guidelines for Addressing Workplace Violence in the Health Sector” by the World Health Organization, the ILO, the International Council of Nurses, and the International Federation of Health Workers. These guidelines are intended to support the development of prevention policies in non-emergency settings and include a questionnaire and research protocol designed to investigate the magnitude and the consequences of violence in these settings.^14^

This initiative has promoted scientific research on workplace violence, enabling a broader understanding of its multiple characteristics and associated factors, which is particularly variable for managers and leaders in the health sector.^14^ This initiative also holds strategic relevance for health care professionals, as it helps guide team-based interventions, fosters safe work environments, and contributes to organizational development, thereby creating favorable conditions for professional practice and staff well-being.^15^

There is scientific production addressing workplace violence; however, studies specifically focused on PHC remain scarce, making it difficult to fully understand the issue at this level of care. Access to accurate and current data supports the development of more effective preventive actions in this setting. The aim of this integrative review is to identify and summarize the available scientific evidence on workplace violence among PHC workers, considering its prevalence, associated factors, consequences, and preventive measures.

## METHODS

This integrative review was conducted using a systematic methodological approach to ensure rigorous organization, synthesis, and integration of scientific evidence. The process included topic identification and research question development; definition of objectives and search strategies; selection of Descriptores en Ciencias de la Salud (DeCS) and Medical Subject Headings (MeSH); database selection; execution of the search according to predefined criteria; implementation of filters and inclusion and exclusion criteria; assessment of methodological quality; and, finally, analysis and synthesis of the findings.

The research question was developed using the SPIDER format. The sample (S) consisted of PHC workers; the phenomenon of interest (PI) was workplace violence; the design (D) included quantitative, qualitative, and mixed approaches; the evaluation (E) focused on the prevalence of workplace violence, associated factors, consequences, and preventive measures; and research type (R) comprised primary and secondary studies published in scientific journals. Accordingly, the guiding research question was: what does the scientific evidence report regarding the prevalence, associated factors, consequences, and preventive measures related to workplace violence among PHC workers?

The literature search was conducted between July 2024 and March 2025 on the Web of Science, PubMed, Scopus, SciELO, and Virtual Health Library databases. The DeCS and MeSH terms “workplace violence,” “primary health care,” and “health personnel” were employed, combined using the Boolean operator AND. The search query used was: ((workplace violence) AND (primary health care) AND (health personnel)). Filters were applied for language (Spanish, English, and Portuguese), full-text availability, and publication period from 2020 to 2025.

The review included original studies with primary data, as well as reviews or synthesis of previous research using quantitative, qualitative, or mixed methods, that addressed workplace violence among PHC personnel. Books, reviews, letters to the editor, duplicate studies, and theses were excluded.

**Figure 2:**
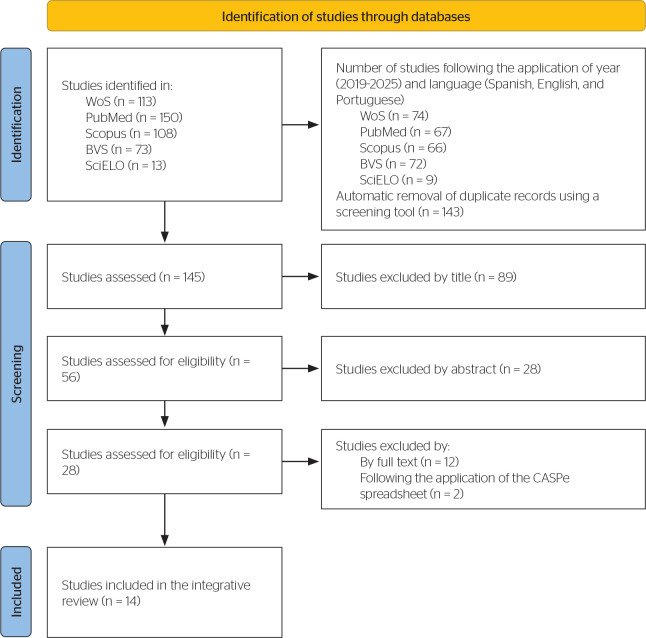
Search and selection flowchart.

The study selection followed the Preferred Reporting Items for Systematic reviews and Meta-Analyses (PRISMA) guidelines (Figure 2). A total of 457 articles were retrieved from the databases, and titles, abstracts, and full texts were screened based on predefined inclusion criteria. Screening was performed using the Rayyan platform, reducing the sample to 16 articles. For full-text appraisal, the Critical Appraisal Skills Programme Español (CASPe)^16^ instrument was applied to evaluate the methodological quality of the studies. Only those with adequate internal validity and relevance, according to the corresponding checklist items, were included, resulting in a final selection of 14 studies.

BVS = Virtual Health Library (Biblioteca Virtual en Salud); WoS = Web of Science.

A data extraction matrix was created to compile general information from each study, such as country, design, target population or sample, and context. A cross-analysis and thematic coding process was then conducted, grouping the findings on workplace violence among PHC workers into four main dimensions: prevalence, associated factors, consequences, and preventive measures (Table 1). This process enabled the integration of results from quantitative, qualitative, and mixed studies and the identification of similarities and differences.

**Table 1:** Characteristics and findings of the selected studies

Country and year	Sample	Context	Prevalence	Associated factors	Consequences	Preventive measures
Brazil 2020^17^	106 physicians, nurses, nursing technicians/assistants and CHWs	17 Family Health Units of PHC in Porto Alegre, Brazil	Verbal aggression: 65.1% Bullying: 14.2% Racial discrimination: 10.4% Physical assault: 8.5% Sexual harassment: 4.7%	Patients: verbal aggression (79.4%) and bullying (46.7%) CHA and physicians: aggressions among co-workers	Remaining overly alert, vigilant, and tense Absenteeism Desire to leave the profession	NM
Spain 2020^18^	22,748 health care professionals (physicians, nurses, nursing assistants, and other professionals), of which 10% worked in PHC settings	PHC facilities in the autonomous community of Aragon, Spain	24.6% reports of workplace violence 31.4% of verbal violence 3.4% of physical violence	Being a physician Being female Having fewer years of professional experience	NM	NM
Spain 2020^19^	5,587 reports from physicians, nurses, nursing assistants, midwives, social workers, and administrative personnel	Madrid Health Service: 89% from PHC and 11% from SHC.	89% prevalence of workplace violence Verbal violence: 75.4% Physical violence: 4.4% Threats 54.1% Coercion: 24.8%	Being female Being a nurse Being a patient: 60.6% (main perpetrator)	Alterations in health status were reported in 94.8% of PHC workers, but these alterations were not specified.	NM
Saudi Arabia 2020^20^	360, 180 each from PHC centers (physicians, nurses, pharmacists, technicians, clerks, and others)	15 PHC centers in Dammam and Al Khobar, Saudi Arabia	46.9% prevalence of workplace violence Verbal violence: 90% Bullying: 34.3% Physical violence: 3%	Working the morning shift Little awareness of violence reporting system Perceiving the reporting system as not efficacious Being a patient or a patient’s companion (main perpetrator)	The majority of participants reported that nothing occurred Decreased performance Feeling guilty Absenteeism	NM
Turkey 2020^21^	752 participants (physicians, nurses, midwives, medical secretaries, laboratory technicians, X-ray technicians, and data managers)	PHC units in the province of Antalya, Turkey	Subjected to mobbing: 16.8% Partially subjected to mobbing:13.6%	Being female Being a nurse-midwife Being in a managerial position (perpetrators) Being between 36-45 years of age (the most victimized group in both genders)	NM	NM
Brazil 2021^22^	169 PHC workers (nurses and nursing technicians/assistants)	1 public hospital and 26 basic health units	51% prevalence of workplace violence:	Being a TENS Frequent contact with patients Showing concern about workplace violence Being a patient, followed by a boss and a co-worker (main perpetrator)	NM	NM
Kuwait 2021^23^	446 participants (220 physicians, 226 nurses)	50 PHC centers in five health regions in Kuwait	Prevalence of physical and psychological workplace violence: 89% (6.4% experienced physical violence, and 77.3% experienced psychological violence): Verbal abuse: 48.8% Bullying/mobbing: 10.1% Sexual harassment: 1.9% Racial harassment: 20.3%	Being a nurse Working full-time Working in shift Working between 6 pm and 7 am	NM	Security measures Improved surroundings Restrict public access Patient screening
Brazil 2022^24^	647 health care professionals, of which 449 (69.4%) worked in PHC: nurses, nursing technicians/assistants, physicians, dental surgeons, oral health assistants, oral health technicians, and CHWs	PHC units in 23 municipalities and one reference hospital in the state of Santa Catarina, Brazil	22.41% prevalence of workplace violence	Being a nurse and/or nursing assistant Having a chronic disease Negative perceptions regarding recognition at work Interpersonal relationships at work assessed as indifferent	Remaining extremely “super alert” Feelings that the activities became more painful Avoiding thinking and talking about the episode Presenting memories, thoughts, or images of what happened	NM
Saudi Arabia 2022^25^	288 physicians and nurses	PHC centers in Buraidah city, in Qassim region, Saudi Arabia	41.2% prevalence of workplace violence Verbal violence: 98.2%.	Working the morning shift: 88.1% Inside the workplace: 96.4% Being a patient: 79.7% Being a female: 63.2% Being between 21 and 45 years of age: 72.1% Greater professional experience Unmet service demand Overcrowding Long waiting time	NM	Security guards Mandatory penalty for offenders Implementation of appointment system for patients
Brazil 2023^26^	11 nurses and nursing technicians/assistants	Three PHC units in the city of Ribeirão Preto, Brazil	All participants suffered violence in their workplace at some time in their professional practice	Being a patient (main perpetrator) Lack of medical appointments available and of appointments without prior patient booking	Psychological: feelings of incompetence regarding their work or distancing themselves from what happened Organizational: greater staff transfer to another service, not taking on certain care tasks or changing them	Surveillance cameras Security agents Group spaces for professional training Role of government in raising awareness on the role of health professionals Participation of the Regional Council of Nursing
Malasia 2023^27^	23 studies (16 quantitative, 4 qualitative, and 3 mixed), from countries across Asia, Europe, and North and South America	The topic of interest concerned every category of PHC personnel	Verbal abuse: 46.9-90.3% Threats or assault: 13-44% Bullying: 19-27% Physical violence: 15.9-20.6% Sexual violence: 2-17%	Female gender Male gender “Reserved” and “careless” personality types Less work experience Being a nurse of physician Area of lower socio-economic status Working in operational services involving contact with people (customer service, home visits)	Psychological: anxiety, stress, or burnout Emotional: feeling guilty, ashamed, and punished	Carrying a personal alarm Leadership Task management Situational awareness Decision-making
Spain 2023^28^	659 self-completed forms administered to physicians, nurses, dentists, midwives, and social workers, among others)	PHC Management of Tenerife, Spain	Prevalence of workplace violence: 72.5% Verbal violence: 90.8% Physical violence: 1.3% Verbal and physical violence: 7.9%	Being female Being a nurse Being a patient (main perpetrator)	NM	Security personnel Security cameras Panic button
Turkey 2024^29^	303 family physicians	Family Health Centers of 11 central districts in İzmir, Turkey	A total of 82% stated that they had been subjected to violence throughout their professional life	Being female: 89.9% Being a patient or patients’ relative (main perpetrators) Low income (perpetrator) Low educational level (perpetrator)	A total of 40.8% expressed “losing interest in their profession”	Legal regulation Increase in criminal penalties Improving communication Provide additional training Balancing work and personal life Hiring more staff Reducing patient workload Addressing social and economic issues
China 2025^30^	568 primary care physicians	PHC institutions in the city of Chengdu, China	44.3% prevalence of workplace violence in the preceding year Emotional abuse: 38.0% Threats: 24.5% Physical assault: 9.8% Verbal sexual harassment: 5.5% Sexual abuse: 1.6%	Higher education levels were associated with increased odds of experiencing workplace violence Being a patient: 81.6% Being a patient’s relative: 44.2%	Low work efficiency: 56.2% Decreased patient trust: 32.7% Anxious feelings: 32.7%	NM

CHW = community health worker; NM = not mentioned; PHC = primary health care; TENS = higher-level nursing technicians (técnicos en enfermería de nivel superior).

## RESULTS

The studies included in this integrative review, originating from various countries and cultures, examined workplace violence in PHC. Cross-sectional quantitative designs predominated, along with some qualitative and mixed studies. The populations encompassed different occupational categories, especially nurses, higher-level nursing technicians (técnicos en enfermería de nivel superior, TENS), and physicians. The reported prevalence of workplace violence in PHC ranged from 22.4% in Brazil to 89% in Kuwait. Verbal violence or verbal abuse were the most frequent form, with rates between 31.4% and 98.2%, whereas physical violence appeared less often (1.3% to 9.8%). Episodes of bullying, sexual harassment, and racial discrimination were also identified, though less frequently.

The most commonly reported factors included female gender, working as a nurse or TENS, direct contact with patients, and morning shifts. In certain contexts, physicians showed higher prevalence, revealing variations according to professional role and organizational environment. Individual consequences involved psychological effects such as stress, anxiety, guilt, shame, and self-doubt regarding professional competence. At the organizational level, absenteeism and increased staff turnover were reported. Preventive measures were scarce and focused on surveillance strategies, such as security cameras, guards, and communication training; only six studies addressed them in detail.

## DISCUSSION

This integrative review shows that the prevalence of workplace violence in PHC ranges from 22.4% to 89%, with verbal violence or abuse being the most frequently reported form.^17^,^24^,^26^,^27^ This trend is consistent with findings from hospital-based studies,^31^-^33^ suggesting that workplace violence is pervasive across different levels of care. One study estimated that approximately 63% of health care personnel experience verbal abuse in their workplace,^34^ which may be explained by the direct and continuous contact with patients, as well as the normalization of aggressive behaviors in the work environment, facilitating the expression of frustration through insults or shouting.^35^,^36^ Physical, racial, and sexual violence were reported less frequently,^17^,^20^,^27^ likely because they occur in less visible or private settings, which makes reporting and documentation more difficult. However, hospital-based reviews show a significant prevalence of sexual violence,^37^,^38^ highlighting contextual differences.

Among the associated factors, working as a nurse or nursing technician (TENS) is linked to a higher risk of workplace violence,^22^,^24^,^28^ a finding consistent with studies conducted in Asia and Europa.^39^,^40^ Hence, in certain contexts, such as that reported Serrano et al., physicians may be more vulnerable,^18^ reflecting how occupational roles influence exposure. Direct and continuous contact with patients or service users under stress or anxiety, particularly during morning shifts, increases the likelihood of verbal aggressions.^20^,^25^,^41^-^43^

Sex plays an important role as well: women predominantly experience psychological and verbal violence, whereas men tend to report more physical aggresions,^18^,^19^,^21^,^28^ consistent with the findings of Jatic et al.^44^ This pattern may be linked to the feminization of the health care workforce and the perception of women as less vulnerable, making them targets of subtle or concealed forms of aggression,^45^,^46^ as well as to cultural associations of men with strength and authority. Structural factors, such as prolonged waiting times and lack of available appointments, are likewise associated with increased workplace violence.^25^,^26^ Feng et al.^47^ confirm that unmet needs and long waits are major drivers of violence, contributing to user frustration that is then directed towards health care personnel.^22^,^26^,^28^,^48^,^49^ Additionally, violence may originate from coworkers, physicians, or managers, reflecting dynamics of power and hierarchy.^17^,^21^,^50^

The consequences extend to both individual and organizational levels. At the individual level, reports include stress, anxiety, intentions to leave the profession, loss of interest,^20^,^24^ as well as guilt and shame, reduced performance, and hypervigillance.^17^,^26^,^27^,^30^ At the organizational level, violence is associated with staff turnover, absenteeism, task abandonment, and decreased work efficiency, which affects team stability, continuity of care, and service quality,^13^,^51^ Wang et al.^52^ corroborate these findings by demonstrating the relationship between violence, emotional exhaustion, and intention to resign.

With respect to preventive measures, although they are addressed directly in only a few PHC studies,^23^,^25^-^29^ several strategies have been identified, including surveillance measures, such as surveillance cameras and guards, organizational actions, such as appointment systems and increased personnel, and training initiatives focused on communication and leadership. Evidence suggests that improving communication^53^ and providing continuous training^54^,^55^ son accessible and effective interventions; however, further research is needed on preventive strategies specifically tailored to PHC.

Taken together, the findings illustrate the complexity and multifactorial nature of workplace violence in PHC, highlighting the interaction of individual, organizational, and contextual factors, consistent with the Chappell & Di Martino’s model.^9^ There is a clear need for stronger evidence to support effective preventive interventions that ensure safe, respectful, and violence-free work environments, aiming for zero violence in health services.

## CONCLUSIONS

Workplace violence in PHC is a frequent and multifactorial phenomenon, with verbal abuse being the most common form, especially among nurses and nursing technicians. Individual and organizational factors, such as sex, professional category, direct interaction with patients, and structure of work shifts, contribute to its occurrence, while its consequences affect both workers’ psychological well-being and the efficiency and stability of health care teams.

Although preventive strategies related to surveillance, organizational measures, and training have been identified, their implementation remains limited, underscoring the need for in-depth studies of workplace violence in PHC and for the development of preventive strategies appropriate to this level of care. Creating safe work environments requires not only further research but also concrete institutional actions. This integrative review provides updated information on the prevalence, associated factors, and consequences of workplace violence, offering useful background for guiding future research and the development of preventive strategies and policies that strengthen safety and quality of care in the PHC setting.
